# Price Fairness of Processed Tomato Agro-Food Chain: The Italian Consumers’ Perception Perspective

**DOI:** 10.3390/foods10050984

**Published:** 2021-04-30

**Authors:** Antonella Samoggia, Giulia Grillini, Margherita Del Prete

**Affiliations:** 1 Department of Agricultural and Food Sciences, University of Bologna, Viale Fanin 50, 40137 Bologna, Italy; margherita.delprete5@unibo.it; 2 Faculty of Science and Technology, Free University of Bozen-Bolzano, Piazza Università, 1, 39100 Bolzano, Italy; giulia.grillini@natec.unibz.it

**Keywords:** tomato, price, fairness, consumer, agriculture, food, food chain, system dynamics

## Abstract

Food consumers are increasingly searching for emotions and values when purchasing and consuming food. They search for products that ensure social and environmental sustainability, in addition to more common extrinsic product attributes, such as price, packaging, origin, and brand. In particular, there is increasing interest towards product price fairness. The current study aims at exploring consumers’ perception and understanding of price fairness, focusing on the processed tomato products agro-food chain. The study interviewed 832 people. Data were collected through an online questionnaire with the support of Qualtrics software, and data elaboration was carried out with Statistical Package for Social Science (SPSS). The elaboration includes an Exploratory Factor Analysis (EFA) to identify existing latent factors in the consumers’ perception of enabling agro-food system elements influencing farmers’ reception of fair prices. Then, factor mean values were cross-analysed with socio-economic characteristics and processed tomato consumption habits with Analysis of Variance (ANOVA). Results support the idea that consumers are limitedly aware of the processed tomato agro-food chain dynamics and consider farmers as the most unfairly remunerated partner. Women and frequently purchasing consumers of processed tomato products believe farmers should be treated more fairly. There is a difference between what consumers perceive as fair price distribution and actual price distribution among processed tomato chain actors. Further studies may focus on how fairness attribute impacts on consumer purchasing behaviour.

## 1. Introduction

Tomato is one of the most important crops in the Italian agro-food sector, and it is a key ingredient in the national culinary tradition. In 2019, Italy was the third highest producer of processed tomatoes in the world, with an annual production of around 5 million tons of raw material, after California (13 million tons) and China (5.6 million tons) [1]. Around 75% of world tomato consumption is fresh tomato and 25% is processed tomato, with consumption ratios differing among regions and eating habits. New tomato-producing countries, such as China, have entered the market in the last years. They have carried out significant investments in this food industry product. In a few years, China has turned into a relevant international competitor, threatening the dominant position of the two main country producers, the USA and Italy [2].

In Italy, there are two main tomato-for-processing production areas, that is North and Centre-South regions. The production is fairly equally distributed between the two areas, but North and Centre-South regions have different specializations. The North area produces mainly sauce (36.6%), paste (32.5%), puree (28.6%), whereas the Centre-South area is the leader in the production of sauce (48%), peeled tomato (44%), and puree (8%) [3].

The importance of the processed tomato industry is confirmed by the steady increase in processed tomato consumption in the past twenty years. The highest consumptions of processed tomato products are in Europe, Pacific-Asia region, and in the USA, with between 20 kg and 28 kg per capita per year [4]. Other countries, such as Canada, Japan, and Tunisia, are increasingly including tomato products in their eating habits [4]. Each country has specific drivers of processed tomato market expansion, based on cooking traditions, food trends, availability, and price [4]. Two factors are of major importance in the development of worldwide consumption: Demographic growth and individual consumption. Over the past two years, the development of mature processed tomato markets, such as North America, Brazil, Asia-Pacific, EU15, and Far East, has been driven by demographic growth in addition to an increase in per capita consumption. For the “emerging” markets, such as Russia, Africa, and United Arab Emirates, the main market development driver has been the pro-capita consumption, which has been almost “explosive” [4].

Processed tomato consumption is common in a high number of countries. Consumers appreciate various processed tomato characteristics, including taste and versatility in various Western and ethnic cuisines, leading to colourful food recipes. Yet, processed tomato consumers at global level are exposed to a media coverage often portraying tomato production and harvesting as example of agro-food system unfairness, in particular, due to unfair labour conditions and unfair revenue distribution at the expenses of farmers [5,6,7,8,9,10,11,12]. This paper stems from the awareness that all chain actors, including retailers, processors, farmers, workers, consumers, and public institutions can contribute to an adequate standard of living and working conditions of actors in agro-food chains. However, there is still a need to conceive how each chain actor can actively contribute to a fair price distribution among tomato chain actors. In particular, there is a need to better understand what the consumers’ perception on food chain fairness is. When selecting food and spending monetary resources, consumers have the capability to actively shape the processed tomato chain functioning.

Thus, the aim of the research is to explore the consumers’ perception of processed tomato price fairness and distribution within the chain. The study focuses the investigation on the following issues: (i) the consumers’ consumption and purchasing habits of processed tomato, with focus on frequency, importance of quality attributes, place of purchase,, and importance of promotions; (ii) the consumers’ perception of the revenue distribution in the processed tomato agro-food chain, comparing real versus fair distribution; (iii) the perception about the role of the different actors of the processed tomato agro-food chain in farmers’ price share.

## 2. Literature Review

### 2.1. Conceptualization of Fairness

The concept of fairness has aroused interest, especially in 2008, when milk farmers went on strike against the low milk price for producers [13]. The debate on fairness and fair price distribution is still heated. However, the academic literature is still struggling in the provision of a consistent understanding of the concept of fairness [14,15,16,17,18]. 

The concept of fairness resembles the fair-trade conceptualisation and certification, which guarantees producers’ fair remuneration and working conditions. Fair trade applies to developing countries, excluding producers from European Union (EU) countries [13,14]. The academic research has extensively explored the Fair-trade certification from the consumer purchasing and consumption behaviour perspective, often in connection with premium price marketing practices and willingness-to-pay exploration [19,20]. However, there are few studies about the consumers’ understanding of fairness and knowledge of the agro-food pricing mechanism along the agro-food chain.

The literature presents different theoretical approaches to fairness. Commonly, it is related to people’s aversion to inequity [13] which means giving up something material if the outcome is more equitable [21]. According to Gielissen and Graafland [22], perceptions of price fairness seem to be, first and foremost, related to compensatory justice. Compensatory justice concerns the just way of compensating people for what they lose. A fair price can be seen as a compensation that is equal to the loss suffered by the person being compensated. However, perceptions of price fairness often include distributive concerns beyond merely compensatory concerns. For example, Gielissen et al. [22] find that price increases are judged to be fairer when they benefit poor or small agents than when they benefit rich or large agents. Therefore, it is important to investigate several concepts of distributive justice rather than merely compensatory justice in the perceptions of price fairness. Distributive justice is concerned with the fair distribution of society’s benefits and burdens. Compensatory justice can be interpreted as one particular form of distributive justice. The concept of distributive justice is, therefore, more general than the concept of compensatory justice. The distributive justice formal principle (traditionally attributed to Aristotle) supports that equals should be treated equally and unequals unequally [22].

**Figure 1:**
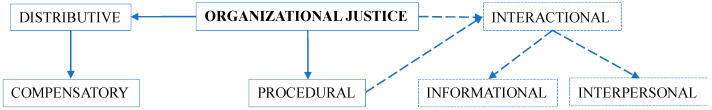
Conceptualization of fairness in literature.

Bush and Spiller [13] integrate distributive fairness with procedural and interactional fairness. Procedural fairness [23] represents the perceived fairness of decision-making processes. Interactional fairness [24] is the behaviour of the trading partner in terms of honesty, respect, quantity, and the quality of information. This perspective does not clarify whether interactional fairness must be seen as an autonomous fairness dimension or as an aspect of procedural components (Figure 1).

Several fairness studies [25,26,27] identify interactional fairness as a third dimension of fairness. However, there are also studies measuring interactional justice as the social part of procedural fairness [28,29]. This perspective does not clarify whether interactional fairness in two other components, that is interpersonal and informational fairness, must be seen as an autonomous fairness dimension or as an aspect of procedural considerations. Colquit et al. [24] divides interactional fairness into two other components: Information and interpersonal justice. These represent the justice perceived, respectively, in the exchange and use of information, and in communication between individuals. The last component of fairness is represented by commitment, which represents the commitment of resources to strengthen relationships and improve supply chain performance.

A key concept in the fairness conceptualisation is the “dual entitlement” (DE). Consumers and sellers recognize each other’s entitlement according to the terms of some reference transaction: Consumers to a reference price, sellers to a reference profit [18,30,31,32]. On one side, the price is considered fair if the price completely compensates the company’s cost and possible cost increases. The fair price is also consistent with fairness norms not to pass cost decreases to the customer, since, in this case, the seller’s profit increases without violating the consumer’s reference price entitlement. Thus, the DE principle states that it is fair that the sellers pursue a pricing rule of raising prices when their costs increase but not reduce their prices when costs decrease.

On the other side, consumers evaluate prices with the belief that while the firm is entitled to a profit, the consumer is also entitled to a fair price. Consumers’ fairness perception refers to the extent to which product attributes, such as the food product price, are judged appropriate and fair. The assessment of price fairness is carried out by comparing a price with a reference price. Reference prices are a key factor in understanding consumer decisions. They serve as the standard to evaluate observed prices. The reference price level is influenced by both internal factors, such as the memory of the last price paid [33] or their most frequently paid price, and contextual, external factors, such as promotions. Internal reference is referred to as “expected or fair price” [34]. Thus, consumers’ reference prices are updated whenever observed prices and reference prices vary [6]. Internal reference prices are stored in people’s memory. Within the DE principle, a price increase that violates the consumer’s entitlement to the reference price, will be considered acceptable only if it is perceived to be necessary to protect the seller’s reference profit. This implies that a price increase that rises the seller’s profit beyond its reference entitlement will be deemed unfair. What is the sellers’ reference fair profit from the consumer perspective is still to be fully conceptualised.

### 2.2. Processed Tomato Attributes and Consumer Behaviour

The agro-food products’ perceived value is increasingly associated by a multidimensional basket of attributes. Some tangible quality dimensions can be easily identified at the place of purchase, whereas the intangible quality dimensions may be credence attributes not associated to purchasing and consumption experience [35]. According to Nelson [17], three fundamental types of food quality attributes can be distinguished:

- Search attributes: They are the visual attributes of the product quality that can be directly observed before the purchase. Examples are appearance (dimension, colour, defects), price, and brand.
- Experience attributes: They are the product quality attributes evaluated during the consumption, such as taste, that generally are not known before the purchase.
- Credence attributes: They are the belief attributes related to the product’s characteristics and production process appreciated by the consumers, such as product origin and sustainable certifications.

In the case of credence attributes, consumers tend to obtain information through undirect indicators. These can be information reported on the label, certification made by external institutions, and trustworthiness of the brand and the retailer [36,37,38,39,40]. On the one hand, the quality of the product and of the chain production process increasingly drives consumers’ choices, on the other hand, the purchasing attitude is highly dependent on the information provided to the consumer. The food product label is an instrument for conveying important information, and consumers may search experience and credence attributes when selecting food product [36,38,41,42,43]. Reliable labels allow the consumer to take an informed purchasing decision that better satisfies its preference. Furthermore, certifications of third parties may not be fully appreciated by consumers, as they do not have the capability to verify the authenticity of those attributes [44].

#### 2.2.1. Colour

Colour is the first sensory attribute consumers check to judge the acceptability of fresh and processed food products. If the colour does not meet expectations, consumers react negatively to the product. The fresh tomato colour is perceived as an indicator of freshness, ripeness and taste. The processed tomato colour is a product attribute providing information on the ripeness and the taste intensity [45,46,47]. The characteristics of the raw tomato for processing influence the quality of the processed tomato final product. Mature tomatoes are the favourite for tomato processing, as they have better flavour and bring more intense-red colour [44,48,49]. The processed tomato quality is assessed by valuing the colour of the heated and cooked processed tomato (i.e., on pizza or pasta) and its velvety texture stability [44,50,51,52,53]. High dilution and not uniformed colour with orange spots are signs of low-quality processed tomato. Past research support that consumers have specific processed tomato colour range expectations that drive the acceptance [47]. As supported by Frez-Muñoz [50], the colour for canned whole peeled tomatoes is the key intrinsic quality attribute most valued by consumers.

#### 2.2.2. Taste

The tomato taste is mainly attributed to soluble sugars, organic acids, and volatile compounds. The importance of taste for consumers is supported by research studies exploring consumers’ appreciation of the processed tomato acidity level in different countries. Sweetness level and product taste, respectively for Chilean and for Dutch consumers, are the most important attributes for purchasing processed tomato [50]. This finding is confirmed by other studies, confirming expected taste is a key attribute during processed tomato purchase [51]. The analysed studies confirmed this finding for consumers’ appreciation of greenhouse tomato production. This type of tomato production allows a wide off-season availability of tomato products, but a generally perceived lower appreciation of its flavour. The consumer dissatisfaction towards this type of tomato products’ flavour and taste generates an increasing demand for higher quality products [50]. Other studies confirm the importance of taste for processed tomato consumers. In particular, according to the study of Zhu et al. [52], consumers appreciate tomato juice information related to taste review ratings. Consumers were willing to pay a premium price for tomato juice as the taste review score increased. Consumers believe that the taste reviews are helpful in making the decision about which product to purchase [52].

#### 2.2.3. Price

Price is the second most important factor that affect consumers’ decision when purchasing processed tomato, after freshness [54]. Further studies explored processed tomato-based products, such as ketchup. The study of Meyerding et al. [38] explored German consumer purchasing and consumption behaviour of ketchup attributes. Findings support that processed tomato consumers strongly appreciate low prices, as much as the local origin. More than one fourth of participants were defined as “price-conscious consumers”, as they thought that price is the key product attribute, followed by the origin, and the production method [55].

#### 2.2.4. Packaging

Processed tomato products are sold in several types of packaging, thus providing opportunities for consumers to choose the package that best suits their needs. One of the functions of packaging is communication, to permit the identification of a product, to assist in sales through marketing, and to provide consumers with information about the product use and handling. Packaging plays an important role in attracting consumers’ attention and influencing their purchase decisions through marketing strategies. In this context, a product package can be used as a source of information and recognition, in addition to suggesting extrinsic quality and brand image [55]. According to the literature, consumers’ processed tomato favourite packaging attributes are: The easy-open device, packaging material (brick and glass are perceived as more sustainable than thin), and packaging colour (red is the favourite colour) [50,55,56].

#### 2.2.5. Origin

Research studies support that the local origin of food products plays a fundamental role in consumers’ purchasing decision of processed tomato. In the study of Meyerding [38], German respondents chose the favourite attributes of various processed tomato-based products, including origin, production method, and price. The main findings of the choice-based conjoint analysis revealed that the majority of the respondents preferred the product labelled as “local” food. Similar results were achieved by the Frez–Muñoz study [50] that underlines consumers’ importance for the processed tomato origin in the different countries. The Italian consumers considered the certification of origin as fundamental for their processed tomato purchase decision. Other countries’ respondents consider the origin of limited importance. Thus, the tomato raw material origin may be valued differently in the various countries.

## 3. Materials and Methods

### 3.1. Data Collection

The data collection was carried out with an online survey with the support of Qualtrics, an online data collection software. The survey was open during Summer 2020. It was launched on different online platforms on the internet, and personal and social media, such as Facebook, WhatsApp, and Instagram of the researchers’ Universities. The previous questionnaire-testing phase allowed the researchers to fine-tune unclear questions and develop a robust data collection instrument. The data collection process was monitored on a regular basis by the researchers. When the consumer response was slowing down, the survey promotion campaign was re-publicised. The data collection ended when researchers observed that the promotion campaign was progressively yielding lower number of responses.

The questionnaire was structured based on an extensive literature review on the research topic (Table 1 and Appendix A). The survey was divided into five sections aiming to identify consumers’ information on: First, habit of processed tomato consumption; second, perception of importance of processed tomato attributes; third, perception of revenue distribution among processed tomato chain actors; fourth, perception of enabling agro-food system elements influencing farmers’ reception of fair prices; finally, consumers’ socio-economic information.

### 3.2. Sample

The survey was filled in by 832 consumers. Data cleaning yielded a final convenience sample of 810 questionnaires used for data analysis. The sample included mostly women (67%), and respondents had an average age of 28 years old, were Italian mainly coming from a wide range of North Italian regions, had a level of education fairly equally distributed among high school (47%) and university degree (46%), and a family income below 55,000 € (84.2%) (Table 2).

### 3.3. Data Analysis

Data analysis followed four steps. First, a descriptive analysis was carried out in order to understand the socio-economic characteristics and consumption habits.

Second, to measure the consumers’ perception of revenue distribution among processed tomato agro-food chain actors, consumers stated what is the estimated price share distribution (S estimated), and estimated fair price share distribution (S fair) of each processed tomato agro-food chain actor, that is farmer, processor, and retailer. These values were then compared to the real price share distribution (S real) of processed tomato agro-food chain actor. The real price processed tomato farmer shares derived from farmers’ association expert analysis. For each share estimations, that is estimated real share and estimated fair share distribution, consumers provided estimated percentages for each of the agro-food chain actors, including farmer, processor, retailer. Percentage values had to be between 0% and 100%, and the total had to be 100%. Then, the research explored (i) the consumers’ perception of the estimated price share compared to the real price share, as follows: S real minus S estimated; (ii) the consumers’ perception of the fair price share compared to the estimated price share, as follows S fair minus S estimated; (iii) the consumers’ perception of fair price share compared to the real price share, as follows S fair minus S real.

Third, the research aimed at identifying the consumers’ perception of the enabling agro-food system elements influencing farmers’ reception of fair prices. Consumers were provided with a set of statement-items (Appendix A). Consumers provided their rating for each of the items with a 7-point Likert scale (1 = not at all important, 7 = totally important). The initial items were twenty-three. Thus, the data analysis aimed at identifying existing latent factors in consumers’ perception of the enabling agro-food system elements influencing farmers’ reception of fair prices. Then, it applied the principal components methods (PCA) and the Varimax rotation. Three items had factor loadings below 0.5, and thus were excluded. The remaining twenty items’ factor loading value was above 0.556 and grouped into six components. Given the limited number of missing values in the variables included in the factor analysis, and in order to strengthen the elaboration results, the listwise method was adopted. The choice of factors was made on the basis of the Eigenvalue criterion being higher than 1, as well as considering the cumulated variance explained by the factors taken together. The cumulated variance explained by the six factors was 63.58%. Factor-based scores were calculated using the Bartlett score as a refined method [62,63]. The factors were saved as new variables by calculating their mean value. The values of Cronbach Alpha (CA) were calculated to test the reliability of the factors. The CA values range between 0.749 and 0.823. Past studies [64] set the following classification of CA values: “>0.9—excellent, >0.8—good, >0.7—acceptable, >0.6—questionable, >0.5—poor, and <0.5—unacceptable”. Thus, the present research CA values are good.

In the last phase, data analysis aimed at understanding if consumers’ socio-economic characteristics and processed tomato purchasing and consumption habits influence consumers’ perception of the enabling agro-food system elements influencing farmers’ reception of fair prices. Factor mean values identified in the third phase of data analysis were cross-analysed with socio-economic characteristics and processed tomato purchasing and consumption habits, using ANOVA analysis. Factor mean values were dichotomized as above versus below 4 within the 7-point Likert scale. The values of the variables with significant ANOVA tests were calculated to capture how consumers’ socio-economic characteristics and processed tomato purchasing and consumption habits influenced consumers’ perception of the enabling agro-food system elements influencing farmers’ reception of fair prices. Data elaboration was performed with the support of the software SPSS (IBM, version 26, Armonk, NY, USA).

## 4. Results

### 4.1. Processed Tomato Consumption Habits

Consumers eat processed tomato products maximum twice per week (55%), mostly purchase them at hyper or supermarkets (69%), and the majority purchase them, sometimes, when on promotion (66.5%). Processed tomato long shelf-life favours a consumer purchasing behaviour oriented towards food products stocking (Table 3).

### 4.2. Products’ Attributes That Drive Processed Tomato Purchasing Behaviour

Consumers expressed the level of importance of product characteristics and attributes when purchasing processed tomato (Table 4). The most important attribute was the taste (mean 6.2), followed by the geographical origin of the raw material (mean 5.2), the price of the pack (mean 4.8), and the brand (mean 4.2). Product’s packaging and promotion seem to be minor attributes in the choice of processed tomatoes (mean 3.7). Standard deviation values support there is consistent consumers’ perception of the relevance of taste (std. dev. 1) and price (std. dev. 1.3) among key processed tomato products’ attributes.

### 4.3. Fair Revenue Distribution in the Agro-Food Chain from Consumer Perspective

The research provides insights on the consumers’ views on the price shares received by the processed tomato product agro-food chain actors (Table 5). Results support that the consumers overestimate the farmer share (S estimated = 6.5% compared to S real = 14%). Moreover, according to the consumers, farmers should gain an additional share of 22.9% compared to the real share they receive to achieve a fair distribution. The estimated perceived share for the processors was very close to the real one (S real = 38% versus 38.4%). According to the respondents, in a fair distribution, food retailers should lose 16.2% of their revenue. The retailer share is underestimated (S estimated = 41.1% compared to S real = 48%). The farmers and food retailers are the agro-food chain partners with the highest deviation between the estimated shares and the fair shares. Consumers’ responses support that these two actors should compensate each other. In a fair distribution, food retailers would lose the 16.2% of their revenue, processor would lose 6.7%, and farmers may increase their share up to +22.9%.

### 4.4. Consumers’ Perception of Farmers and Price Fairness

The research aimed at better understanding consumers’ understanding of fairness in the processed tomato agro-food chain. The research focused on what interventions and initiatives agro-food system actors, from the farmers to the policy makers, should adopt to ensure higher levels of fairness, and, in particular, on farmers’ fair price. The research explored what understanding consumers have on fairness constructs, in terms of fair price distributions, procedures, and interactions, and if fairness theories can support a better conceptualisation of the price distribution. The factor analysis carried out lead to the definition of six factors describing consumers’ perception of fairness constructs. The identified six factors are (Table 6):

- Interactional fairness: This construct identifies the behaviour of the trading partner in terms of honesty, respect (interpersonal fairness), quantity, and quality of information (informational fairness). Consumers believe that all processed tomato agro-food chain actors can mutually contribute to ensure higher revenues for farmers. This factor merges concepts identified in past studies [13];
- Procedural fairness: This construct merges the views that farmers’ fair price is achieved if minimum standards of subsistence, coverage of production costs, and common price level for farmers are ensured. This factor items combine past studies understanding of procedural fairness [13] and the farmers’ rights [22]. It confirms the approach based on positive rights ethics of Shue [65], who argues that a minimum of subsistence is a basic right.
- Farmers’ management: This construct merges items that provide a view of respondents’ perception of farmers’ working performance. This factor highlights the role of farmers’ managerial skills, inclination towards investments, efforts, commitment, flexibility, and predisposition to adopt high input performance. These items merge consumers’ views on farmers’ abilities and managerial skills emerging from past studies [58,59,60,61].
- Systemic action: This construct focuses on three chain actors’ responsibility in ensuring that farmers receive a fair price. In particular, farmers’ fair price can be ensured thanks to the other actors’ contribution. Consumers should pay more, and processors and retailers may reduce their profits. This factor expresses an agro-food systemic call-to-action shared among various chain actors.
- Distributive fairness: This factor adopts a comparative approach in understanding fairness towards farmers. Farmers are perceived in a worse-off position, compared to processors and retailers, with specific attention to the risks and challenges farmers are exposed to. The items of this factor confirm past research findings focused on price fairness in the form of revenue distribution along the agro-food chain [13].
- Cost reduction: This factor is a one-item factor stressing the active responsibility that farmers have towards their own managerial practices and cost reduction. Fair price for farmers can be achieved by lowering their production costs, thus entailing higher and sustainable profits.

The factors’ mean values provides insights on the consumers’ fairness perception. The most relevant factors are “Interactional Fairness” (mean 5.6) and “Systemic action” (mean 4.4). Consumers think that retailers, processing industries, politics, and consumers themselves can have a positive role in ensuring a fair price for farmers. Moreover, they think that consumers may pay a higher price. This action must be associated with decreased profits for the processing industry and the retailers. A combined action can lead to higher value for the farmers.

Consumers give limited importance to “Procedural fairness” (mean 2.7), “Farmers’ work” (mean 2.9), and “Production Cost reduction” (mean 2.9). These factors partially affect the farmers’ capability of obtaining fair pricing. Consumers think the actions that favour farmers’ fair pricing are limitedly attributable to farmers’ efficiency, management practices, or intended to deliver farmers’ minimum subsistence. Finally, consumers do not adopt a comparative approach among agro-food chain actors when focusing on farmers’ prices (“Distributive fairness” mean 1.9). The profit of the other actors does not represent a valid benchmark for approaching fairness within the chain with particular attention towards farmers.

Consumers perceive that farmers’ fair prices come thanks to interactional fairness and correct and honest relations among all agro-food chain actors. The responsibility lays within the system, and is the result of a systemic action. Farmers alone should be accountable for the low prices they get, and their farm management practices can only limitedly explain the agro-food system unfairness. The systemic actions may include proactive practices from each chain actor. Consumers may pay more; processor and retailers decrease their profits.

### 4.5. Consumers’ Socio-Economic Characteristics and Consumption Habits

Results support that consumers’ socio-economic characteristics and consumption habits limitedly impact on the perception of price fairness of processed tomato products (Table 7). “Farmers’ work” and “Distributive fairness” are the types of fairness mostly affected, respectively with three and two highly significant values. The characteristics that mostly influence price fairness perception are gender, and consumption frequency.

Results support women believe in interactional fairness more than men (Table 8). They think that “Farmers’ work” and “Distributive fairness” are less important to achieve fair prices for farmers compared to men. Younger consumers are more in favour of “Procedural fairness.” Moreover, consumers with higher academic degrees, and with higher income, think that farmers’ work efficiency impacts on prices fairness. Finally, consumers that eat processed tomato rarely think that “Distributive fairness” and “Cost reduction” may impact on price fairness more than consumers that consume processed tomato more frequently.

To conclude, women, young consumers, with higher level of education and income, as well as consumers with low consumption frequency have more intense opinions. They think that farmers’ price fairness can be achieved intervening at agro-food system level with the contribution of stakeholders.

## 5. Discussion

The most important attribute driving consumers’ processed tomato purchasing behaviour is the taste. The second key attribute of processed tomato purchasing behaviour is the territorial origin of the raw material. These findings confirm past studies’ results [38,50,51,52]. The current study highlights that price is the third key attribute for processed tomato purchasing. Other studies support that price is of key importance, following taste [44]. Consumers perceive price and promotion as two separate product attributes. Price is a key attribute, whereas promotion is rated as the least important. This finding suggests that consumers may not necessarily look for discounted prices or other promotional forms. This is only limitedly consistent with processed tomato market sales data, according to which around half of processed tomato products is sold when on price promotion.

The results on the perception of price distribution support that, according to consumers, farmers receive unfair payments, and should earn a wider share compared to the perceived price distribution. Past studies show that consumers believe that the dominant, industrial food system is not fair [16,66]. In consumers’ view, processors and, especially, food retailers tend to be unfair [67], and should diminish their shares. The overestimation of the share for intermediaries underlines the partial consumer knowledge about food production and agro-food chain organization. This factor confirms past studies’ findings. Bush and Spiller [13] support the idea that consumers underestimate the real farmer share in the milk supply chain. Chang and Lusk point out how consumers found purchasing decisions on inaccurate perceived beliefs [15]. Grashuis supports that the consumers have limited knowledge on chain profit allocation. In particular, disclosed information on profit allocation to farmers, compared to other chain actors, would positively impact on their willingness-to-pay for farmer-owned label [36]. The food retailers are the agro-food chain actors with the highest deviation between the estimated and the fair shares. From a consumers’ perspective, to obtain a fair price distribution for the products investigated, food retailers should lose around 16% of their share [13].

Consumers have a moderately negative perception of the interactional and distributive fairness among chain actors. First, consumers perceive the need of a higher level of interactional fairness. In particular, there is need of honesty, respect, and better quantity and quality of information among the trading partners. Second, consumers perceive the need of a higher level of distributive fairness, as they believe the revenue distribution along the agro-food chain is not fair. These results confirm past studies’ findings [13,18,66]. Consumers believe agro-food chain actors treat farmers dishonestly, and policies should promote an information campaign to support farmers. Such information would benefit consumers by clarifying their possible inaccurate beliefs. These may put consumers at risk of exploitation by some food manufacturers and retailers [15].

Furthermore, consumers perceive the procedural fairness as not crucial. The subsistence and equal prices for all farmers, and the coverage of the production costs are not perceived as key aspects for the achievement of price fairness. This confirms past research findings supporting consumers may fail to take into account the full range of vendor costs, and to over-attribute price differences to profit [66]. Whereas other past research provides a different perspective. They support that ensuring the coverage of production costs and the reliability of prices are important for consumers’ price fairness assessment [13,22]. Other studies sustain that the equal prices and profits for all farmers are limitedly significant for consumers [13], and the seller’s cost structure plays an important role in consumers’ assessing of whether a price, or a price increase, is acceptable or fair [19,66].

As supported by the results obtained in the factors “Interactional fairness” and “Systemic action,” according to consumers, fairness towards farmers is the result of a systemic collaborative intervention of agro-food system stakeholders and policies. Consequently, various actors may play a role in reaching a fair price for farmers. In particular, the option of increasing the consumer price of the product to ensure a farmers’ fair price is not positively perceived. This is consistent with studies raising the attention on the need to monitor fairness initiatives on market prices consumers will pay [68].

The results on the influence of consumers’ socio-economic characteristics and purchase habits on farmers’ fair price perception provides interesting considerations, limitedly explored in past research. Gender and rate of purchase lead to consumers’ different perceptions. Women tend to believe that farmers may be treated more honestly, work efficiently, and receive unfair prices. Consumers purchasing processed tomato products more than twice per week believe farmers’ price is not fair and believe farmers do not have to reduce farmers’ production cost to achieve adequate profit. The importance of purchasing frequency in shaping fairness perception confirms past research [33,34]. In addition, highly educated and high-income consumers believe farmers’ work may be more efficient. Younger consumers aspire to an equal revenue distribution, the need to ensure a fair price to cover the production costs, and to guarantee the farmers’ right to minimum subsistence. Consumers’ socio-economic profile and purchasing habits affect fairness conceptualisation. Women, young, and high-frequency consumers of processed tomato feel the need to strengthen the agro-food chain fairness by safeguarding farmers’ work with an adequate remuneration.

### 5.1. Managerial Implications

The study results provide insights on consumers’ interest in fairer processed tomato product, and in the definition of potential marketing and business strategies in order to achieve a fairer revenue distribution in the processed tomato agro-food chain.

From a marketing management perspective, it is relevant to take into account consumers’ sensitiveness towards fairness and farmers’ sustainability. The promotion of a marketing communication campaign based on agro-food chain fairness concept, and farmers’ economic sustainability would be innovative, and attract new consumer segments. As past studies support, current consumer trends show increasing consumers’ interest towards local, small-scale, and domestic products, even at the expense of organic food market [16,22]. Farmers’ market and local food chains are gaining higher market shares. This is due to various private and public initiatives, as support for short food chains and local economy. Within this emerging trend, fairness and sustainable price distribution within the chain may encounter consumers’ interest.

Consumers may consider themselves as being a weak agro-food chain actor, mirroring farmers’ condition. Direct purchases by processors, cooperatives as farmer-led organization, and fair-trade marketing, both for domestic farmers and farmers in developing countries, could offer marketing benefits. In those initiatives, process control, independence, and distributive advantages for farmers should be highlighted to underline the positive impact of buying these products, especially for domestic markets [13]. As past research supports, consumers have shown an increasing interest in not only how their food is produced, but how is the distribution of benefits resulting from consumers’ food purchases [15].

A further strategy may concern retailers. Retailers may adopt certification schemes to prove managerial practices that favour fairness and chain sustainability. Some of these may focus on small-scale producers [10]. This marketing management practice may strengthen the consumer loyalty towards retailers. It would reinforce consumers’ trust that what the retailers offer comes from fair managerial practices. The aim is to pursue fair competitive managerial practices without disclosing contractual and food chain management practice details that may be beyond consumers’ interest. Moreover, it would counterbalance increasing retailers’ obfuscated pricing strategy. This is based on personalised customer-dependent price setting that prevents consumers observing prices offered to other consumers [69]. This pricing strategy practice strengthens consumers’ inability to distinguish misleading pricing strategies [70], and limits consumers’ capability to define its own reference prices and definition of what is a fair price [30,31,32,33,34].

Finally, agro-food system managers should take into account the increasing consumer’s interest towards agro-food system fairness. They may adopt coordinated management strategies enabling a win-win conditions for the different agro-food actors, with specific attention towards farmers’ and overall agro-food chain’s economic and management sustainability [71]. In addition, preferences and willingness-to-pay could be crucial for the industry in finding the proper market segment and different strategies to penetrate this market [20,36]. As supported by past studies, consumers’ willingness towards fairness premium price is influenced by who receives this premium and how much the consumer earns [36,72], and is dependent on the initial price levels [14,36]. When consumers believe there are unfair practices in the agro-food system and they may influence the change, consumers are motivated to make ethical purchasing choices [20].

### 5.2. Policy Implications

Price fairness and agro-food chain sustainability importance has increased in the last years. Recent policies are increasingly addressing the issue. The European Commission document cites “Common Agricultural Policy (CAP) will ensure access to safe, high quality, affordable, nutritious and diverse food” (European Commission, 2019) [73]. This confirms the European Union Treaty statement according to which the CAP will “provide a fair standard of living for the agricultural community” (European Union, 2002) [74].

Tomato is a food product often portrayed by the media and non-profit organisations as reaching consumers’ tables from unfair and unsustainable agro-food chain management practices. The identified unfair practices are the low price paid to farmers by processors, the unfair harvesting labour treatment, and the undefined raw material origin. To tackle some of these aspects, the EU has promoted the extension of the EU fruit and vegetables market observatory activities to include the fresh and processed tomato. Starting from October 2020, the EU develops a tomato-focused report to monitor tomato raw material prices across the EU countries and trade exchanges [75,76]. The increasing importance of fairness issues within the agro-food system is supported by the European Commission consultation open to agro-food system actors on unfair trading practices (UTPs) [77]. The aim is to define a robust baseline in view of the evaluation of UTP Directive [78,79]. The sustainability and fairness of processed tomato production should be achieved through systemic, multi-actor policy initiatives. Agro-food system actors should support practices to favour the agro-food system sustainability. Retailers, processing industries and policies should help farmers get a fairer price for their products.

The current research shows that consumer preference for boosting the position of farmers in the agro-food chain is in accordance with the European Commission political initiatives. Furthermore, fairness cannot be achieved only by increasing the farmer share. It is important to increase the farmers’ negotiating roles when determining the distribution of the price along agro-food chain systems. As supported by past research, policies may help farmers develop food products with a set of consumer-oriented attributes, including fairness, credence, and process properties [14], and should aim at balancing non-monetary and monetary benefits for the farmers [80]. In liberalized agricultural markets, it is difficult for politics to find effective ways to boost farmers’ shares without adverse effects, such as higher prices paid by consumers [68]. There is need for a better organization of farmers by means of production cooperatives to improve agricultural and chain efficiency, and by aggregative organisations for stronger negotiating power [71,81]. These could help countervail retailers’ and processors’ power, and strengthen farmers’ position [13].

### 5.3. Limitations and Future Research

The present study may have some limitations to be, possibly, addressed in future studies. First, the sample includes consumers from a single country and on a single product. This delivers robust and consistent findings on the consumers’ perception analysis, and of a specific product understanding by consumers. However, future studies may extend the analysis to provide a cross-country and multi-product perspective. Second, the sample includes fairly young consumers. This element provides solid sample characteristics, but may create a bias, since, as shown by the literature, younger consumers tend to be particularly sensitive towards sustainability issues. Finally, the survey was carried out only by internet because of the mobility limitations due to the COVID-19 pandemic situation. Future studies may consider integrating the methodological approach with qualitative data collection analysis, such as focus group and face-to-face interviews. This would allow to better capture consumers’ understanding of fairness concept.

## 6. Conclusions

The current study analysed consumers’ perception of fair price distribution and how consumers perceive the concept of fairness in the processed tomato agro-food chain. Farmers are perceived as the most unfairly compensated actor in the agro-food chain. Consumers overestimate farmers’ share. This finding confirms the limited consumer knowledge about food production and agro-food value chain organization. From a consumer point of view, a fair distribution should foresee that the highest share for processed tomato should remain in farmers’ hands, whereas retailers should be the chain actor to have the lowest share. In order to increase consumer awareness on tomato chain fairness, it would be useful to share information on what farmers, as well as all other chain actors, receive. The price and economic information should be enriched with evidences on the managerial practices of the agro-food chain. The educational and awareness-raising communication campaign may increase consumers’ understanding and critical approach to fairness within the agro-food chain functioning.

## Figures and Tables

**Table 1:** Literature sources of the questionnaire.

Questionnaire Section	References
Processed tomato consumption habits	[14,44]
Perception of processed tomato attributes	[38,44,45,46,47,48,49,50,51,52,53,54,55,56]
Perception of revenue distribution among processed tomato chain actors	[13,14]
Perception of farmers and price fairness *	[13,22,57,58,59,60,61]
Socio-economic information	[14]

* Appendix A provides detailed literature review references of this section questionnaire items.

**Table 2:** Socio-economic characteristics of the respondents.

Socio-Economic Characteristics	% of the Total
Gender	
Male	32.3
Female	67.0
Other	0.7
Total	100
Age	
18–24	57.6
25–34	26.3
34–45	5.0
over 45	11.1
Total	100
Nationality	
Italian	94.8
other EU countries	4.2
non-EU countries	1.0
Total	100
Region	
Trentino-Alto Adige	38.1
Emilia-Romagna	29.2
Veneto	11.2
Lombardia	4.9
Others	16.6
Total	100
Level of education	
junior high school	0.3
high school	46.6
university diploma	46.1
post-university degree	7.0
Total	100
Annual family income	
<15,000 €	12.0
15,001–28,000 €	33.7
28,001–55,000 €	38.5
55,001–75,000 €	10.0
>75,000 €	5.6
Total	100

**Table 3:** Processed tomato consumption and purchasing habits of the respondents.

Habit	Typology	% of the Total
Processed tomato consumption	Never	1.5
	1–2 times/week	55.0
	3–4 times/week	35.5
	5–6 times/week	6.8
	7 times/week or more	1.8
	Total	100
Purchase organic processed tomato	yes	46.0
	no	54.0
	Total	100
Location of purchase	Hyper and Supermarkets	68.5
	Discount retailers	19.9
	Directly from farmers	6.2
	Small retail shops	5.4
	Total	100
With product promotion (discounted price, buy-one and save-more, with coupon, etc.)	Never	12.6
	Sometimes	66.5
	Often	19.7
	Only	1.2
	Total	100

**Table 4:** Products’ attributes that affect consumer purchasing behaviour of processed tomato products.

	Mean	Std. Dev.
Taste	6.2	1.0
Origin	5.2	1.7
Price	4.8	1.3
Brand	4.2	1.7
Promotion	3.7	1.6
Packaging	3.7	1.6

Note: Scale from 1 to 7 (1 = Not at all important, 7 = Totally important).

**Table 5:** Price real shares, estimated shares, and fair shares of the processed tomato product agro-food chain actors (%).

	S Real	S Estimated	S Fair	S Real–S Estimated	S Fair–S Estimated	S Fair–S Real
Farmer	14	20.5	43.4	−6.5	+22.9	+29.4
Processor	38	38.4	31.7	+0.6	−6.7	−6.3
Retailer	48	41.1	24.9	−7.9	−16.2	−23.1
Total	100.0	100.0	100.0			

Note: S real as from literature; S estimated as perceived by consumers; S fair as perceived by consumers.

**Table 6:** Result of the exploratory factor analysis.

Questionnaire Items	Factors					
	Interactional Fairness	Procedural Fairness	Farmers’ Work	Systemic Action	Distributive Fairness	Production Cost Reduction
Farmers receive a fair price:						
- compared to the profit gained by processing industry					0.784	
- compared to the profit gained by retailers					0.861	
- considering the risks and challenges they encounter					0.754	
*Cronbach’s alpha 0.792*						
Farmers receive a fair price:						
- if the price covers the production costs		0.801				
- if the price ensures the farmers’ right to minimum subsistence		0.861				
- if is the price that all the farmers get		0.720				
*Cronbach’s alpha 0.823*						
Farmers receive a fair price:						
- if consumers are willing to pay higher prices				0.607		
- if processors diminish their profits				0.813		
- if retailers diminish their profits				0.831		
*Cronbach’s alpha 0.749*						
Consumers should ensure farmers receive a fair price	0.698					
Agricultural policies should ensure farmers receive a fair price	0.684					
Food retailers should ensure farmers receive a fair price	0.810					
Food processors should ensure farmers receive a fair price	0.825					
Politics must promote an information campaign	0.694					
*Cronbach’s alpha 0.814*						
Farmers should lower production costs						0.798
Farmers make low investments in technology and innovation			0.584			
Farmers do not put enough effort in their work			0.556			
Farmers have low management skills			0.856			
Farmers have low flexibility in decision-making			0.826			
Farmers select input providers with limited focus on input performance			0.763			
*Cronbach’s alpha 0.805*						
Mean values of factors	5.6	2.7	2.9	4.4	1.9	2.9

**Table 7:** Consumers’ socio-economic characteristics and consumption habits.

	Interactional Fairness	Procedural Fairness	Farmers’ Work	Systemic Action	Distributive Fairness	Production Cost Reduction
Gender	0.000 ***	0.430	0.000 ***	0.443	0.001 ***	0.220
Age	0.500	0.001 ***	0.767	0.724	0.110	0.723
Education	0.808	0.109	0.001 ***	0.923	0.626	0.467
Income	0.553	0.923	0.006 ***	0.280	0.921	0.231
Consumption frequency	0.176	0.097	0.093	0.136	0.002 ***	0.004 ***
Organic	0.053	0.141	0.203	0.950	0.022 **	0.238
Place of purchase	0.569	0.818	0.760	0.971	0.020 **	0.979
Promotion	0.673	0.681	0.204	0.713	0.432	0.188

Note: **, *** Significant at *p* < 0.05; *p* < 0.01. Variables were dichotomized as follows: Gender: F versus M; Age: Below versus above average age (28 years); Education: With versus without academic degree; Income: Above versus below 28 k family/year; Consumption: Low frequency versus medium-high frequency (twice/week); Organic: Consumers purchasing organic processed tomato versus consumers not purchasing organic processed tomato; Place of purchase: Big retailers versus small retailers; Promotion: Consumers purchasing processed tomato on promotion versus consumers not purchasing processed tomato on promotion.

**Table 8:** Significant mean values of the socio-economic characteristics and consumption habits versus the factors of price fairness.

Socio-Economic Characteristics		Factors				
		Interactional Fairness	Procedural Fairness	Farmers’ Work	Distributive Fairness	Production Cost Reduction
Gender	Men	5.4		3.2	2.1	
	Women	5.7		2.8	1.8	
Age	<28 year-old		2.8			
	>28 year-old		2.4			
Education	without academic degree			2.8		
	with academic degree			3.1		
Income	<28,000 Euro/year			2.8		
	>28,000 Euro/year			3.0		
Consumption	<2 times per week				2.0	3.1
	>2 times per week				1.8	2.8

## Data Availability

The data that support the findings of this study are available from the corresponding author upon request.

## Appendix A

	**Source**
Farmers receive a fair price	
compared to the profit gained by processing industry	[13]
compared to the profit gained by retailers	[13]
considering the risks and challenges they encounter	[57]
if the price covers the production costs	[13]
if the price ensures the farmers’ right to minimum subsistence	[22]
if is the price that all the farmers get	[13]
if consumers are willing to pay higher prices	[22]
if processors diminish their profits	[22]
if retailers diminish their profits	[22]
Consumers should ensure farmers receive a fair price	[13]
Agricultural policies should ensure farmers receive a fair price	[13]
Food retailers should ensure farmers receive a fair price	[13]
Food processors should ensure farmers receive a fair price	[13]
Politics must promote an information campaign	[13]
Farmers should lower production costs	[22]
Farmers make low investments in technology and innovation	[58]
Farmers do not put enough effort in their work	“effort” is a recurring and key word in all studies analysed
Farmers have low management skills	[60,61]
Farmers have low flexibility in decision-making	[60,61]
Farmers select input providers with limited focus on input performance	[59]
